# Social Support, Socio-Economic Status, Health and Abuse among Older People in Seven European Countries

**DOI:** 10.1371/journal.pone.0054856

**Published:** 2013-01-30

**Authors:** Maria Gabriella Melchiorre, Carlos Chiatti, Giovanni Lamura, Francisco Torres-Gonzales, Mindaugas Stankunas, Jutta Lindert, Elisabeth Ioannidi-Kapolou, Henrique Barros, Gloria Macassa, Joaquim F. J. Soares

**Affiliations:** 1 Centre of Socio-Economic Research on Ageing, Italian National Institute of Health and Science on Aging, I.N.R.C.A., Ancona, Italy; 2 Scientific Direction, Italian National Institute of Health and Science on Aging, I.N.R.C.A., Ancona, Italy; 3 Centro de Investigación Biomedica en Red de Salud Mental (CIBERSAM), University of Granada, Granada, Spain; 4 School of Public Health, Griffith University, Gold Coast, Queensland, Australia; 5 Department of Health Management, Lithuanian University of Health Sciences, Kaunas, Lithuania; 6 Department of Public Health Science, Protestant University of Applied Sciences, Ludwigsburg, Germany; 7 Department of Sociology, National School of Public Health, Athens, Greece; 8 Department of Hygiene and Epidemiology, Medical School, University of Porto, Porto, Portugal; 9 Department of Public Health Sciences, Institution for Health Sciences, Mid Sweden University, Sundsvall, Sweden; 10 Division of Social Medicine, Department of Public Health Sciences, Karolinska Institute, Stockholm, Sweden; Cardiff University, United Kingdom

## Abstract

**Background:**

Social support has a strong impact on individuals, not least on older individuals with health problems. A lack of support network and poor family or social relations may be crucial in later life, and represent risk factors for elder abuse. This study focused on the associations between social support, demographics/socio-economics, health variables and elder mistreatment.

**Methods:**

The cross-sectional data was collected by means of interviews or interviews/self-response during January-July 2009, among a sample of 4,467 not demented individuals aged 60–84 years living in seven European countries (Germany, Greece, Italy, Lithuania, Portugal, Spain, and Sweden).

**Results:**

Multivariate analyses showed that women and persons living in large households and with a spouse/partner or other persons were more likely to experience high levels of social support. Moreover, frequent use of health care services and low scores on depression or discomfort due to physical complaints were indicators of high social support. Low levels of social support were related to older age and abuse, particularly psychological abuse.

**Conclusions:**

High levels of social support may represent a protective factor in reducing both the vulnerability of older people and risk of elder mistreatment. On the basis of these results, policy makers, clinicians and researchers could act by developing intervention programmes that facilitate friendships and social activities in old age.

## Introduction

Social support is defined in terms of social network characteristics such as assistance from family, friends, neighbours and other community members. It involves “social transactions the aims of which are to assist individuals in coping with everyday life, and particularly in responses to critical situations” [1].

Hall & Wellman [2] proposed the concepts of social network and social support as mediating constructs towards well-being, to explain how various social exchanges among individuals, mainly in situations of need, may influence health outcomes. Further studies refer to the health benefits resulting from social support due to its capacity to reduce the risks for both physical and cognitive illnesses [3], [4]. According to Krause [5], received support is the amount of tangible help provided by social network, whereas perceived support is the subjective evaluation of the received help. Perceived support is a crucial resource when stress is experienced [6], and for individuals with limitations in daily living activities (ADLs, i.e. everyday routine activities generally involving functional mobility and personal care, including eating, bathing, dressing, toileting, walking and control of continence) [7]. Higher levels of interpersonal trust also appear to be positively correlated with good self-assessed physical health and mental well-being [8].

The evidence suggests that social support depends on several demographic/socio-economic and geographical factors. In general, the perception of available support is higher among younger and married persons, and persons with higher socio-economic and employment status [9]. Concerning gender, some studies find major informal perceived support for women [10], whereas others report larger health benefits from social networks for men [11], [12].

Concerning the elderly, social support may represent a main source of personal care and well-being [13], and the aspects already emphasized in the general context of social support become more critical and amplified by the various problems connected to an ageing population. Social vulnerability, which is a concept related to a low social support, is indeed higher among people with individual frailty, and it increases with age. Greater social vulnerability is associated with mortality in older adults [14]. Very old age is moreover associated with lower levels of income [15], and reduced social networks and social support are more frequent among older people with low socio-economic position [16]. Very old age is also associated with lower levels of health [17]. The positive influence of social support on the health of the elderly is well documented; in particular emotional support from offspring is positively associated with a higher degree of well-being, and less distress and cognitive impairments among older people without a spouse [18]. Conversely, loneliness in old age has been suggested to be a risk factor for morbidity and mortality [19]. In particular, an absence of informal support can have a serious impact on health and quality of life of low-income elderly women living alone, and this may also lead to premature institutionalization [20]. Further, recent findings emphasise the importance of family and friendship for healthy aging [21], and confirm that chronic stress and loss of functions in older people may be mitigated by informal and formal support [22]. Family solidarity, in its affective aspect, can indeed be considered a “robust concept” and a fundamental element for social integration in old age [23].

Social isolation and a low level of social support may be crucial risk factors for elder abuse, besides older age, chronic health conditions and cognitive deficits. Also, if the old person is economically dependent this can add to the burden and stress experienced by family caregivers of older relatives and play a role in elder abuse. A systematic review of studies on the prevalence of elder abuse and neglect in various countries [24] has reported abuse rates ranging between 3.2–27.5% in the general population. In this context, social isolation may represent a crucial dimension of social insecurity and vulnerability affecting older people due to their minor (or lack of) role in society. When this is combined with a reduced independence in later life, it may expose older persons to mistreatment and/or to neglect. Isolation thus appears to be a risk factor for all forms of elder abuse (e.g. physical). Consistent correlations between different types of elder abuse and low social support [25], and also consistent positive associations between most subtypes of mistreatment and depression [26] have been found. In contrast, a high level of social support may represent one potential protective factor for elder mistreatment [27]. Greater levels of social support can modify and reduce depression in old age as a risk factor for elder abuse, mainly in women [28].

Scrutinizing this latter topic in Europe is very important as few studies have addressed elder abuse and its features from a comparison perspective, within multi-cultural and multi-national contexts [24], [29]. The aim of this study was to determine whether social support (perceived help from family, friends and significant other) was related to various selected dimensions in old age in seven European countries, including the crucial aspect of elder abuse. We hypothesized that a high level of social support would be associated with increased health and well-being, and negatively linked with the risk of mistreatment. Lack of social support, in a context of dependency and vulnerability, may indeed represent a potential risk of exposure to abuse.

## Materials and Methods

### Data Sources/Collection and Ethics Statement

The present study is based on the data from the ABUEL survey (Elder Abuse: A multinational prevalence survey) carried out to investigate the prevalence and risk factors of violence against the elderly in seven urban centres within selected European countries: Ancona (Italy), Athens (Greece), Granada (Spain), Kaunas (Lithuania), Ludwigsburg (Germany), Porto (Portugal) and Stockholm (Sweden). The data were collected cross-sectionally among community-dwelling elderly populations during January-July 2009 by face-to-face interview or interviews/self-response. All survey materials (e.g. the questionnaire) were translated into the relevant languages, back-translated, and culturally adapted. Interviewers in each country were carefully instructed about the administration of the questionnaire and ethical behaviour. Strong emphasis was put on voluntariness and confidentiality of participation.

Sampling and administration procedures were carried out according to the national, ethical and legal requirements for this type of studies. The potential participants were informed about the study by means of a letter explaining aims and contents of the ABUEL project. Written informed consent from participants, regarding also their anonymity, rights and freedom to stop the interview at any moment, was obtained prior to data collection. Ethical permission/approval also was sought and received in each participating state from the national/university or regional ethics review boards. Greece was an exception.

In detail, the full names of the ethics committees/institutional review boards were the following: Regional etisk kommittee vid Karolinska Institutet (Karolinska Institute, Regional Ethics Committee) in Sweden; Ethikkommission des Landes Baden-Wuerttemberg (Ethics Committee of the State of Baden-Wuerttemberg) in Germany; Comitato di Bioetica INRCA, Istituto Nazionale di Riposo e Cura per Anziani, Ancona (National Institute of Health and Science on Aging, Bioethics Advisory Committee) in Italy; Kauno regioninio biomedicininiu tyrimu etikos komitetas (Kaunas Regional Research Ethics Committee) in Lithuania; Comité de Ética do Hospital de João, Porto (Ethics Committee of the John Hospital, Porto) in Portugal; Comité de Etica en Investigación de la Universidad de Granada (Research Ethics Committee, University of Granada) in Spain. In Greece the field work was carried out by the QED company which is member of ESOMAR that provides ethical guidance through global guidelines, and actively promotes self-regulation in partnership and researchers with a number of associations across the globe. The members, as well as their company contact details, are listed in the ESOMAR Members Directory. Members undersigned, and agreed to abide by the ICC/ESOMAR International Code on Market and Social Research, which has been jointly drafted by ESOMAR and the International Chamber of Commerce.

The final sample (sex and age-stratified) included 4,467 persons (2,559 women, 57.3%) randomly selected (registry/census based) from the general population, except for Greece and Portugal. In Greece a sampling by random route of the elderly was obtained, according to a walking scheme that allowed selecting elder persons in households, and in ‘Open Care Community Centres’ (KAPI). In Portugal a cluster sampling method was used, and subjects were recruited among the members of a cohort (EPIPorto) of urban dwellers previously selected using random digit dialling. The inclusion criteria across countries were: (a) women and men; (b) age 60–84 years; (c) not suffering from dementia, or other cognitive impairments, assessed by means of the Mini-Cog [30]; (d) having legal status (national citizens or documented migrants); (e) living in the community (own/rented houses) or homes for elderly (e.g. sheltered houses). The sample size calculation was based on municipal censuses in each participating city, and on an expected abuse prevalence of 13% derived from a recent systematic review [24]. Assuming this prevalence rate, with a precision of 2.6%, a sample size of 633 individuals in each city was required, but considering the infinite population assumption a maximum of 656 individuals was allowed. The sample size was adapted to each city according to the population of individuals aged 60–84 years (representative and proportional to sex and age). Mean response rate was 45.2% across countries. More detailed description of materials and methods, sampling strategy and data collection, target population, cooperation, completion and response rates by country, are reported in a separate paper [31].

### Measures

The participants completed a standardized questionnaire with various validated instruments.

*Social support* was measured with the Multidimensional Scale of Perceived Social Support [32]. It consists of 12 questions (graded 1–7), which can be divided into 3 sub-scales, i.e. support from family, significant other and friends. Each sub-scale has been calculated when all related 4 items have been answered. The possible range of each subtotal score is 4–28. Likewise, the total scale has been calculated when all 12 items have been answered. In this case, the possible range of total score (sum all responses) is 12–84. High scores correspond to high social support (sub-scales, total). For this study, the focus was on the total and sub-scales scores.

*Violence was* assessed with 52 questions based on the UK study on elder abuse [33] and the CTS2 [34]. The participants were asked if during the past year they had been exposed to at least one single episode/event of: psychological (11 items), physical (17 items), sexual (8 items) and financial abuse (9 items), including injuries (7 items). The acts of abuse may have occurred once, twice, 3–5, 6–10, 11–20 or >20 times during the past year, or did not occur the past year. In addition, we assessed neglect (e.g. not helped in routine housework) with 13 items where the participants were asked whether they needed help and received it, needed help but did not receive it or did not need help. Data concerning the perpetrator’s main characteristics were also gathered. For this study, the focus was on exposure to the above-mentioned abuse types, excluding neglect.

*Somatic symptoms* were measured with the short version of the Giessen Complaint List [35], consisting of 24 questions (graded 0–4, no complaints-severely affected). The symptoms are organized according to four types, with six questions in each: exhaustion (e.g. tiredness); gastrointestinal (e.g. nausea); musculoskeletal (e.g. pains in joints or limbs); and heart distress (e.g. heavy, rapid or irregular heart-throbbing). The total score amounts to 96, and the sub-total score in each symptom category ranges from 0–24. The higher the scores, the more one is affected (sub-scales, total). For this study, the focus was on the total score.

*Depressive and anxiety symptoms* were measured with the Hospital Anxiety and Depression Scale [36]. This consists of 14 questions (graded 0–3), with seven questions about depression (e.g. I feel as if I am slowed down) and seven about anxiety (e.g. I get sudden feelings of panic). The total score for depression and anxiety is 21 each. A score of 0–7 corresponds to no cases, 8–10 to possibly cases and 11–21 to probable cases. High scores correspond to high depression and anxiety levels. For this study, the focus was on the total scores.

*Health care use* was measured in form of the number of contacts with different types of health care staff (e.g. physician) and health care services (e.g. primary care). Additionally, we assessed the *number of diseases* (e.g. cardio-vascular) from which the elderly were currently suffering. The questions were derived from the Stockholm County Council health survey [37].

Various *demographic and socio-economic* variables such as age, gender, marital status, living situation, habitation, education level, profession, financial support and financial strain were measured. Age was categorized into five-year groups (60–64, 65–69, 70–74, 75–79, and 80–84). Marital status was assessed as single, married/cohabiting, divorced/separated and widow/er. Living situation (as type of relationship to the person living with the interviewee) was classified as alone, only with partners/spouse, with partner/spouse/others (e.g. daughter), without partner/spouse and with others (e.g. daughter). Habitation was assessed as living in an own property, in a rented place, or other (e.g. housing for elderly). Education level was grouped into seven categories: cannot read/write, without any degree, less than primary school, primary school/similar, secondary school/similar, university/similar, other (e.g. art school). Profession was grouped into six categories: managers/professionals/assistant professional, clerical support/sales workers, skilled agricultural/forestry/fishery workers, assemblers/elementary occupations, housewife/husband, and armed forces. Finally, the financial situation was assessed by means of financial support and financial strain. Financial support asked for the main source of income, and was categorised as work income, work pensions (e.g. age and early retirement pension), social/sick-leave/other pension benefits (e.g. sick-leave/unemployment/social support benefits, disability/sick pension), partner/spouse income (e.g. widower pension), and other (e.g. rentals from own capital, including no financial support). Self-reported financial strain (preoccupation with how to make ends meet) was investigated with the following question: “How often are you worried about the daily expenses? *(e.g. for buying food)*” It was measured in a “no/sometimes/often/always” format. A participant was defined as having “financial strain” if she/he chose any response other than “no”. The demographic and socio-economic variables were customised for each country, but similar in content.

### Statistical Analyses

The bivariate relation between social support and categorical variables (e.g. demographics/, socio-economics and abuse) was analysed with the Kruskall-Wallis test with a Bonferroni correction, following a Shapiro-Wilks test to check for the normality of distributions. Associations between social support and numerical variables (household size, healthcare services use, depression, anxiety, somatic complaints, and number of events of abuse) were analysed with the Spearman correlation test. The analyses of the factors associated with abuse were expanded also with regard to each sub-categories of social support (from family, friends and significant other). Multivariate quantile linear regression models, based on median values, were used to examine the interrelations between social support and various variables (independent). The associations between social support and the independent variables were expressed in un-standardized Betas and their standard errors (SEs). Un-standardized Betas were used, despite difficulties in the interpretation of coefficients, because the main aim in the study was to establish associations among covariates and the dependent variable, not measuring them or comparing different coefficients. The choice of using the Beta coefficients and SEs, as outcome measures from the regression models, is also explained by the need to allow comparisons with other similar studies where this sort of data presentation is the most usual. The statistical packages SPSS 15.1 and STATA 11.1 were used to carry out the analyses.

## Results

### Descriptive Statistics

Table 1 provides a full descriptive summary of the demographics and socio-economic characteristics of the sample. The responses from 4,467 participants, besides 57.3% of women (as already highlighted), also put in evidence that 6.0% of them were single and 65% were married or cohabiting, and that 49.6% lived only with a partner/spouse and 24.2% alone. Further, 76% of the interviewees lived in an own habitation, 24.5% had a primary/elementary education and 39.9% a secondary/intermediate one. With regard to the occupation, 27.6% of the sample were managers/professionals, 27.5% were clerical support/sale workers, and 14.9% were housewives. Finally, 65.9% lived on a work pension and 64% declared to experience financial strains.

**Table 1:** Demographics and socio-economic characteristics of the sample.

	Total (n = 4467)	
Variables	N	%
*Country*		
Germany	648	14.5
Greece	643	14.4
Italy	628	14.1
Lithuania	630	14.1
Portugal	656	14.7
Spain	636	14.2
Sweden	626	14.0
*Age (group years)*		
60–64	1124	25.2
65–69	1088	24.4
70–74	961	21.5
75–79	749	16.8
80–84	545	12.2
*Gender*		
Female	2559	57.3
Male	1908	42.7
*Marital status*		
Single	270	6.0
Married/cohabiting	2903	65.0
Divorced/separated	343	7.7
Widow/er	950	21.3
*Living situation*		
Alone	1078	24.2
Only partner/spouse	2208	49.6
Partner/spouse//others^a^	706	15.9
Without partner/spouse – with others^a^	457	10.3
*Habitation*		
Own	3392	76.0
Rental	930	20.8
Other^b^	143	3.2
*Education*		
Cannot read/write	136	3.0
Without any degree	187	4.2
Less than primary school	338	7.6
Primary school/similar	1092	24.5
Secondary school/similar	1782	39.9
University/similar	855	19.2
Other^c^	73	1.6
*Profession*		
Managers/professionals/assistant profess.	1217	27.6
Clerical support/sale workers	1214	27.5
Skilled agricultural/forestry/fishery workers	707	16.0
Assemblers/elementary occupations	570	12.9
Housewife/husband	656	14.9
Armed forces	45	1.0
*Financial support*		
Work	542	12.1
Work pensions	2939	65.9
Social/sick-leave/other pension benefits^d^	243	5.4
Partner/spouse income	627	14.1
Other^e^	110	2.5
*Financial strain*		
No	1605	36.0
Yes	2857	64.0

a = e.g. daughter;

b = e.g. housing for elderly;

c = e.g. art school;

d = e.g. sick pension;

e = e.g. own capital.

### Internal Reliability of Exposure Variables

Reliability, considered as internal consistency of exposure variables across countries in the study, was assessed using the Cronbach’s α statistic. Cronbach α for total social support was.92, and for the three subscales of family, friends and significant other were.90,.94 and.87, respectively. Cronbach α for violence was: for psychological.85, for physical.80, for sexual.76, for financial.64, and for injuries.70. Finally Cronbach α was for somatic symptoms.92, for anxiety.81 and for depression.80.

### Bivariate Analyses

#### Social support by country and demographic/socio-economic variables

As shown in Table 2, participants from Lithuania reported higher mean scores on social support than those of the other countries, with Portugal showing the lowest (*p<.001*). Individuals under 70 years, in particular those aged 60–64 years, scored higher on social support than older participants, with those aged 80–84 years reporting the lowest (*p<.001*). Participants who were male (*p = .023*), but also participants who were married/cohabiting, living only with spouse-partner, highly educated with university degree and owning their housing reported greater social support than their counterparts (*p<.001*). Those who had been managers-professionals and in the armed forces (*p = .001*), had their main financial support from a work pension and did not experience financial strain (*p<.001*) also scored high on social support.

**Table 2:** Social support by country and demographic, socio-economic variables.

	Social support^a^			
Variables	n	Mean	SD	*P* b
*Country*				*<.001*
Germany	580	68.0	14.8	
Greece	642	67.8	15.7	
Italy	610	67.2	12.6	
Lithuania	629	70.4	12.7	
Portugal	650	63.2	12.8	
Spain	635	67.4	16.2	
Sweden	612	67.6	16.4	
*Age (group years)*				*<.001*
60–64	1098	69.1	13.5	
65–69	1064	68.6	14.2	
70–74	935	66.7	14.9	
75–79	727	66.5	15.1	
80–84	534	64.1	16.0	
*Gender*				* = .023*
Female	2493	66.9	15.2	
Male	1865	68.3	13.7	
*Marital Status*				*<.001*
Single	258	58.9	19.0	
Married/cohabiting	2841	70.0	12.1	
Divorced/separated	331	61.3	18.5	
Widow/er	927	64.3	16.6	
*Living situation*				*<.001*
Alone	1038	61.3	18.3	
Only partners/spouse	2161	70.3	12.1	
Partner/spouse/others^c^	693	68.8	12.1	
Without partner/spouse - with others^c^	450	66.3	15.3	
* Habitation*				*<.001*
Own	3332	68.5	13.9	
Rental	887	64.1	16.3	
Other^d^	139	62.2	17.2	
*Education level*				*<.001*
Cannot read/write	135	59.8	19.0	
Without any degree	186	66.5	18.0	
Less than primary school	335	64.3	16.2	
Primary school/similar	1075	66.9	14.5	
Secondary school/similar	1718	68.0	13.8	
University/similar	835	69.8	13.4	
Other^e^	71	66.9	14.3	
*Profession*				* = .001*
Managers/professionals/assistant profess.	1190	69.2	13.5	
Clerical support/sale workers	1171	67.5	14.2	
Skilled agricultural/forestry/fishery workers	694	66.6	14.5	
Assemblers/elementary occupations	563	65.8	16.6	
Housewife/husband	644	66.6	15.4	
Armed forces	45	71.0	10.7	
*Financial support*				*<.001*
Work	532	67.8	14.3	
Work pensions	2859	70.0	13.4	
Social/sick-leave/other pension benefits^f^	234	60.7	16.5	
Partner/spouse income	622	66.4	15.7	
Other^g^	106	65.9	14.5	
*Financial strain*				*<.001*
No	1563	68.7	14.1	
Yes	2791	66.8	14.9	

a = MSPSS, 12–84;

b = Kruskall-Wallis test: *P<.05*;

c = e.g. daughter;

d = e.g. housing for elderly;

e = e.g. art school;

f = e.g. sick pension;

g = e.g. own capital.

#### Correlations between social support, household size and health variables

As shown in Table 3, household size was positively correlated with social support, indicating that the larger the household, the greater the social support received (r = 0.1276, *p*<.05). Conversely, depression(r = −0.2110, *p*<.05), anxiety (r = −0.2911, *p*<.05), and physical complaints (r = −0.2180, *p*<.05), were negatively correlated with social support, whereas no significant correlation was found with the frequency of health care contacts.

**Table 3:** Correlations between social support, household size and health variables.

	Social support^a^
Variables	Correlation coefficients^b^
Household size^c^	0.1276*
Health care services use^d^	−0.0244
Depression^e^	−0.2110*
Anxiety^e^	−0.2911*
Physical complaints^f^	−0.2180*

a = MSPSS, 12–84;

b = Spearmann correlation:

c = number of people in the household;

d = number of health care contacts;

e = HADS, 0–21;

f = GBB 24, 0–96;

* P<.05.

#### Social support by abuse type and injuries

As shown in Table 4, elderly exposed to psychological, physical and financial abuse, and injury, reported significantly lower scores in total perceived social support than their counterparts. In particular, this was more evident among those who sustained injuries (56.3 *vs.* 67.5, *p* = .001), There were no significant differences concerning sexual abuse.

**Table 4:** Social support by abuse type and injuries during the past 12 months.

Abuse type and injuries	Total Social support^a^				Social support from family^aa^			Social support from friends^aa^			Social support from significant other^aa^		
	n	Mean	SD	*P* b	Mean	SD	*P* b	Mean	SD	*P* b	Mean	SD	*P* b
*Psychological* c				*<.001*			*<.001*			* = .001*			* = .001*
No	3494	68.4	14.03		23.8	5.1		20.2	6.9		24.3	4.9	
Yes	864	63.7	16.40		21.5	6.2		19.4	6.9		22.5	5.9	
*Physical* d				* = .001*			* = .001*			* = .138*			* = .001*
No	4246	67.6	14.50		23.4	5.4		20.1	6.9		24.0	5.1	
Yes	112	61.5	18.15		21.1	6.3		19.0	7.3		21.6	6.3	
*Injuries* e				* = .001*			* = .300*			* = .248*			* = .001*
No	4329	67.5	14.56		23.4	5.4		20.0	6.9		23.9	5.1	
Yes	29	56.3	21.04		18.6	7.3		18.3	7.8		18.5	7.1	
*Financial* f				* = .001*			* = .001*			* = .013*			* = .001*
No	4186	67.7	14.42		23.5	5.4		20.1	6.9		24.0	5.0	
Yes	172	62.3	18.51		21.0	6.8		18.8	7.2		22.1	6.6	
*Sexual* g				* = .318*			* = .005*			* = .281*			* = .385*
No	4325	67.5	14.61		23.4	5.4		20.0	6.9		23.9	5.1	
Yes	33	64.1	17.93		19.8	7.7		21.1	7.0		22.8	6.1	

a = MSPSS, 12–84;

aa = MSPSS, 4–28;

b = Kruskall-Wallis test: *P<.05*;

c = e.g. undermined or belittled what you do;

d = e.g. kicked you;

e = e.g. you passed out from being hit on the head;

f = e.g. tried to make you give money, possessions or property;

g = e.g. touched you in a sexual way against your will.

Concerning subscales, family support was significantly perceived to be lower by respondents exposed to all kinds of violence, except for injuries (no significant differences), and the victims of sexual abuse had the lowest significant score (19.8 *vs.* 23.4, *p* = .005). With regard to perceived social support from friends, the differences between abused and non-abused respondents were significant (but less marked than support from family) in the case of psychological and financial mistreatment. The support from significant other was perceived as much lower by respondents exposed to all kinds of violence, except for sexual abuse (no significant differences), and this result was more evident when injuries and physical violence were involved.

### Multivariate Analyses

#### Factors associated with social support

As shown in Table 5, a higher level of social support was independently associated with being from Greece (ß = 4.91, *p*<.001) and Lithuania (ß = 5.56, *p*<.001), married/cohabitant (ß = 5.35, *p*<.01), divorced/separated (ß = 3.24, *p*<.01), widow/er (ß = 6.67, *p*<.001), living with spouse/partner (ß = 5.25, p<.01) or other persons e.g. daughters (ß = 3.26, *p*<.01), living in larger households (ß = 0.69, *p*<.05), frequent use of health care services (ß = 4.51, *p*<.001), and low scores in depression (ß = −7.21, *p*<.001) and physical complaints (ß = −0.06, *p*<.01). A lower level of social support was independently associated with being from Italy (ß = −2.15, *p*<.05) and Portugal (ß = −4.56, *p*<.001), older age (mainly those aged 80–84 years, ß = −2.28, *p*<.05) and male (ß = −1.63, *p*<.01), having a social/sick-leave/other pension as the main source of financial support (ß = −2.98, *p*<.01), and exposure to psychological mistreatment (ß = −2.51, *p*<.001).

**Table 5:** Multivariate quantile (median) linear regression analysis (un-standardized betas/standard error) of the association between social support, country, demographic, socio-economic, violence and other selected variables.

		Social support^a^	
Independent variables	Categories	Beta	Standard error
Country^b^, *Germany* +	Greece	4.91***	1.02
	Italy	−2.15*	0.92
	Lithuania	5.56***	0.96
	Portugal	−4.56***	0.9
	Spain	0.48	1.1
	Sweden	0.3	0.88
Age bands^b^, *60–64* +	65–69 years	−0.47	0.66
	70–74 years	−1.69*	0.72
	75–79 years	−1.06	0.78
	80–84 years	−2.28*	0.89
Gender^b^, *Female* +	Male	−1.63**	0.53
Marital status b, *Single* +	Married/cohabiting	5.35**	1.68
	Divorced/separated	3.24**	1.2
	Widow/er	6.67***	1.07
With whom one lives^b^, *Alone* +	Only partner/spouse	5.25**	1.57
	Partner/spouse/others^d^	2.61	1.76
	Without partner/spouse - with others^d^	3.26**	1.01
Household sizec, e		0.69*	0.27
Main source of financial support^b^, *Work pensions* +	Work	0.23	1.09
	Social/sick-leave/other pension^f^	−2.98**	1.05
	Partner/spouse income	1.07	0.96
	Other^g^	−2.09	1.48
Health care services usec, h		4.51***	1.08
Depressionc, i		−7.21***	0.54
Physical complaintsc, j		−0.06**	0.02
Psychological abuseb, k, *No* +	Yes	−2.51***	0.58
Constant		58.86***	2.98

+ = Baseline;

a = MSPSS, 12–84;

b = categorical variables;

c = continuous variables;

d = e.g. daughter;

e = number of people in the household;

f = e.g. sick pension;

g = e.g. own capital;

h = number of health care visits;

i = HADS, 0–21;

j = GBB-24, 0–96;

k = e.g. undermined or belittled what you do;

* P<.05; **P<.01; ***P<.001.

## Discussion

### Social Support, Country and Demographic/Socio-economic Variables

Drawing from the significant results of the multivariate analyses, we found that social support differed in relation to a wide range of associated factors. Respondents from Greece and Lithuania were more likely to report increased support, whereas the opposite emerged in Portugal and Italy. With regard to geographical differences in the level of perceived social support, some authors [38], [39] observed cross-national/cultural variations. Other studies [40] reported a strong “familistic” cultural tradition in Mediterranean countries, and a greater support from non-family networks in the non-Mediterranean ones. In our study we partly found the abovementioned differences across countries. The positive relation with perceived social support, which is reported from Greeks, could thus be explained by a greater family support available in the Mediterranean Greece, whereas the high rate of perceived social support in Lithuania could be more related to non-family networks in the non-Mediterranean areas. Drawing from some national studies, the Lithuanian context could also be explained by the high level of education of elder participants from this country [41], which is linked in turn to a greater social involvement, comparing with the less educated ones [42]. In Italy and Portugal we found a negative association with the level of perceived social support. We can argue that these two typical Mediterranean contexts, with a strong “family-oriented” connotation, have been negatively influenced by recent and similar demographic/socio-economic changes (e.g. low fertility rates, smaller households, increasing presence of women in the labour market, urbanisation and increasing individualisation). These changes, according with some authors, could possibly account for a dramatic reduction of the traditional resilience of family networks, as primary welfare providers for older people in these areas [43], [44].

Being aged 70–74 or 80–84 years was associated with low social support levels, which is in line with other findings from previous literature confirming that the oldest people are more isolated and less socially supported [45]. The recent and actual demographic changes (different family patterns due to declining marriage, increasing divorce and cohabitation, reconstitutions of new family) indeed highlight significant family disruptions and a decreasing care role of the family. According to literature, this has crucial implications for the provision of social support to older persons, especially to the “oldest old” suffering from health problems and functional limitations [46] thus further affecting the reduction in social relations which takes place especially after retirement [47].

Males were less likely to receive social support than females, and consequently were at higher risk of not being adequately supported by the informal network. An explanation of these results is supported by the literature [48], showing that women have a more socially-oriented life-style, are more concerned about establishing social relations, and appear to receive support from multiple sources, whereas men tend to rely usually on the wife. It is to be considered that in the multivariate analyses (Table 5), whose finding are discussed, men scored negatively (like those living on social sick leave and those aged 70–74 and 80–84 years), and this suggests that men, but not women, experience a lower social support. Conversely, in the bivariate analyses (Table 2) social support is higher for men. This may seem counter-intuitively but it is the result of the adjustment of other covariates in the regression analysis. Likewise, as mentioned in the introduction, the literature on gender and perceived social support is controversial. Some authors refer to major perceived support for women [10], whereas others report that men have larger social networks [12].

Social support from family, friends and significant other was more likely to be received by those who were not living alone but with partner/spouse or other persons. Additionally, being married/cohabitant, divorced/separated and widowed were associated with higher levels of social support. These findings suggest that living arrangements, as the effective presence of a spouse/partner or other persons in the household, more than marital status (with the exception of single individuals) are predictors of stronger social support. Many studies have shown that married/cohabiting persons can count on spousal/partner social support and that the spouse/partner is usually the principal figure providing help [49], whereas not having the assistance of a spouse/partner is a significant predictor of living alone [50].

Predictably, household size was found associated with increased social support levels. In this respect, previous studies have stressed that household size may have various implications for the well-being of older people. Some authors suggested that social support itself can be seen as resulting from certain structural/quantitative characteristics of the social network, e.g. size and composition of one’s interpersonal ties [2], [51]. Other studies found that larger households may improve intergenerational solidarity and support, and also reduce isolation in later life [52], thus buffering poor health and improving quality of life [53].

Social-sick-leave/other pension benefits as the main source of financial support was associated with low social support levels. This is in line with authors [54] indicating that respondents experiencing a poor economic situation and low occupational status, such as unskilled workers, report the poorest social support. This also confirms what previous studies have asserted, i.e.the perception of available support is higher among individuals with higher occupational status and higher income levels [55], whereas reduced social support is more frequent among socio-economically disadvantaged older people [16].

### Social Support, Depression, Somatic Complaints and Health Care Services

In our study depressive symptoms were associated with decreased social support. Our results concerning depression confirm previous studies showing that strong social support may be associated with increased mental health, and it may act as coping resources in older age [47], whereas individuals who have more restricted networks are most likely to exhibit signs of depression [56]. Literature also shows that social support may exert a buffering effect reducing the impact of depression on functional status [57], and that support from marriage, in particular, protects against worse psychological health over time [58].

Further, somatic complaints were associated with low levels of social support. Our findings are consistent with those from recent studies emphasising a positive association between health and social support, and thus an overall protective effect of social networks on disability may be supposed. Literature on the topic highlights that this was especially true for older individuals with chronic disease, and for those suffering from stress associated with ADLs limitations [59], whereas social isolation may also increase the risk for coronary heart disease events and mortality [60]. Further authors showed that social support may also provide health benefits to older persons by facilitating recovery when ill or by protecting against illness through biological mechanisms [61]. Family members’ actions may indeed often support the recovery of their older relatives by providing intangible resources and emotional help [62].

Concerning physical and mental health on the whole, many studies have shown that social support has an important impact on the health and well-being of older people, but due also to the lack of a theoretical framework useful for understanding this type of effect [63], the way it works is not clear [64], i.e. social isolation could also be a symptom of physical/mental health problems rather than a cause [65]. In any case, our findings are in line with above-mentioned research indicating that “deficiencies” in social support have a negative impact on health.

Our findings indicate also a positive relation between frequent use of health care services and increased social support levels. These results seem in line with studies indicating that frequent contacts with the social network and higher levels of social support are associated with greater use of general medical services [66], whereas conversely socially isolated older adults use fewer health services and seem to have small social networks resources [67]. Our findings seem thus to follow the ‘bridging’ mechanism [68] which indeed predicts a positive association between service use and social support (i.e. social networks bring individuals into contact with health services when needed). Therefore, our findings are not in line with Cantor’s ‘hierarchical-compensatory’ mechanism [69], i.e. when social support is unavailable from the social network, the request for formal services increases.

The combination of the results of this section - frequent use of health care services was independently associated with increased social support, whereas depressive symptoms and somatic complaints were associated with decreased social support – indicates, on the whole, relations concerning the dimensions investigated. We are aware that, as the data are cross-sectional, they don’t allow the establishment of causal links between variables (but only hypothesis of relations), and thus no causal inference can be made in this regard. Anyway, our results seem to suggest that older individuals who report higher levels of social support, also use health care services, showing less physical complaints and low levels of depression,. It could also be hypothesized that the lack of social support exerts a negative influence on cognitive functioning and physical health in later life, especially when health care services are not, or very little, used. Greater levels of social support in old age might therefore be considered to reduce the risk for physical-functional diseases and mental illnesses, and mitigate depression.

### Social Support, Abuse Type and Injuries

Only having been exposed to psychological abuse was independently associated with decreased social support. This finding indicate that greater levels of social support might exert a protective influence against the risk of psychological abuse in older age, a phase of life which is too often marked by a weakening of one’s social networks. These results are consistent with those of other studies revealing that generally emotional and affective support and solidarity from family might be more important than instrumental support for psychological symptoms [23], [70]. Literature in particular highlights that, on the one hand, the experience of social support involves such things as good intimate social relations (spouses/partners), feeling cared for, valued and being part of a network of relationships, with a resulting sense of well-being [4]. Psychological abuse, on the other hand, involves among other things harsh and insulting words, threats, silent “treatment” and being ignored [33], [34], which seem to be the opposite of central components in social support. Thus, this may explain the relationship between psychological abuse and a low level of social support. It is worth noting that, according to many authors, social isolation, loneliness and low levels of social support seem to co-exist with elder abuse, but more as risk factors for violence than causes [25], [71], [72]. Apparently in our data low social support may be both a cause and effect of abuse, at least for psychological one. Low levels of social support may indeed create a potential violent emotional context, but psychological abuse itself could also lead to the perception of reduced social support.

### Limitations

This study has some limitations. First, the generalizability of the findings can be questioned. Data are only from large urban centres in seven European countries and are based on self-reports by older participants, thus being subject to possible recall bias. Moreover, the study excluded elderly with cognitive impairment (not able to appropriately complete the survey). Second, the data are cross-sectional thus not allowing the establishment of causal links between variables. Third, the relatively low numbers of participants who reported some types of abuse episodes (e.g. injury and sexual abuse) suggests caution in the interpretation of findings, and this doesn’t allow further inferences or generalizations. It is also very likely that this could be linked with a systematic under-reporting of abuses. Future research in this area will require a longitudinal design in order to test the correlation found between social support and other dimensions, elder abuse included. Despite these limitations, our study provides the following benefits: cross-national data on various aspects of elder abuse (e.g. social support); a workable definition of abuse (including injuries) and validated instruments to assess the phenomenon; findings and tools which could be used by policy makers, clinicians and researchers at the European and country levels for a range of activities (e.g. monitoring abuse, awareness campaigns).

### Conclusions

Our study highlights the multiple connections between social support and various dimensions in later life. Variations and similarities between countries, concerning cultural attitude in relationships across Europe, were shown. Further, factors related to increased perceived social support were found, such as living in larger households and not alone, frequent use of health care services, low scores in depression and in physical complaints. A lower level of social support was conversely associated with older age, male gender, having a poor economic situation, and exposure to psychological mistreatment. High levels of social support may thus represent a key factor in reducing and preventing the vulnerability and isolation of older people, and the risk of elder mistreatment, at least emotional. On the basis of these results, policy makers and clinicians could act by developing adequate intervention programmes, which aim at promoting opportunities for older people to engage in social activities. Moreover, further more broadly and longitudinal research is needed to explore causality and direction of the relations found. In this respect, our findings confirm previous results and contribute with additional evidence, thus suggesting further directions and implications to investigate more in depth the impact of support systems on the life of frail elderly.
